# You Are Not Alone: A Serial Mediation of Social Attraction, Privacy Concerns, and Satisfaction in Voice AI Use

**DOI:** 10.3390/bs13050431

**Published:** 2023-05-20

**Authors:** Tae Rang Choi, Jung Hwa Choi

**Affiliations:** 1 Department of Strategic Communication, Texas Christian University, Fort Worth, TX 76109, USA; t.r.choi@tcu.edu; 2 Department of Communication, University of South Alabama, Mobile, AL 36688, USA

**Keywords:** artificial intelligence (AI), voice AI, smart speaker, loneliness, social attraction, privacy concerns

## Abstract

The popularity of voice-activated artificial intelligence (voice AI) has grown rapidly as people continue to use smart speakers such as Amazon Alexa and Google Home to support everyday tasks. However, little is known about how loneliness relates to voice AI use, or the potential mediators in this association. This study investigates the mediating roles of users’ perceptions (i.e., social attraction, privacy concerns, and satisfaction) in the relationship between users’ social loneliness and intentions to continue using voice AI. A serial mediation model based on survey data from current voice AI users showed that users’ perceptions were positively associated with behavioral intentions. Several full serial mediations were observed: people who felt lonely perceived (1) voice AI as a more socially attractive agent and (2) had fewer privacy concerns. These aspects were each tied to satisfaction and subsequent usage intention. Theoretical and practical implications are discussed.

## 1. Introduction

Recent years have seen a rise in the popularity of voice-activated artificial intelligence (voice AI); such devices are expected to occupy more than 400 million households worldwide by 2025 [1]. Voice AI enables machines to understand human speech through natural language and a human-like voice [2]. Smart speakers such as Google Home respond to voice commands, enabling users to perform tasks ranging from setting alarms to engaging in small talk. These devices’ convenience has contributed to their popularity, given that users can perform tasks hands-free. Smart speakers are a form of interactive media: their roles as conversational partners can foster an interactive environment [3]. Research on interactive media partly focuses on how such devices affect people psychologically (e.g., in terms of loneliness). Mental states have been shown to influence people’s adoption of interactive technologies [4,5]. Documented associations exist between loneliness and the use of smartphones (i.e., for texting and calling) and social media [5,6,7]. Smart speakers are a relatively new type of interactive media that facilitates voice-based user interaction [3]. However, little is known about how user’s social loneliness shapes voice AI use.

Loneliness is a common experience among the general population. Loneliness warrants scholarly attention: more than half of U.S. adults are reportedly lonely, and this mental state represents a worldwide problem [8,9]. Loneliness is an unpleasant emotional state arising, either quantitatively or qualitatively, from deficient social relations and a discrepancy between craved and actual social connections [10,11]. When one’s social needs are not met, loneliness and other feelings compel one to fulfill these needs [12]. Technology continues to permeate modern society. Interactive communication media may help ease negative moods. Indeed, lonely people tend to use social media to compensate for inadequate offline connections [6,13,14]. In a similar vein, recent research [15] has found that social interaction is a major driver of smart speaker use. Technological advances may enable smart speakers to function as companions for people experiencing loneliness. Voice AI could even serve as an alternative to social media given the negative effects of social comparison on these networking platforms [7].

Machines have already been framed as communication partners. Research on AI and human–computer interaction has explored the social aspects of voice AI based on a sound theoretical framework: computers are social actors (CASA) [2,16]. CASA posits that individuals interact with machines as if they were social agents because people tend to mindlessly apply social attributes to them with minimal social cues [17,18,19]. In terms of social signal processing, interdisciplinary studies on artificial agents’ interactive abilities suggest that voice AI’s cues (i.e., human-like dialogue) can induce users’ social responses [2,20]. These social signals produce an interaction between interlocutors. Features such as using natural language to respond to users’ requests create a sense of companionship, because a device is used within proximity [21,22]. Given that proximity and experience of AI can influence how AI can be identified as humankind [23], voice AI may therefore be felt to be an appealing device for lonely people.

User perceptions have previously been studied and found to be associated with the use of interactive media. Privacy concerns (worries about one’s personal information) are prevalent in users’ perceptions of interactive media [24]. Overhearing users’ conversations and collecting personal data raise related concerns around voice AI. Possible misuse of personal information partly informs users’ satisfaction with and adoption of smart speakers [25,26]. Scholars have also attended to voice AI’s social attraction, namely the extent to which users perceive these devices as socially attractive communication partners [27]. Voice AI’s ability to engage in small talk renders the technology socially appealing. Indeed, people who use voice AI for conversations tend to view the device as a friend [15]. It is thus reasonable to argue that lonely people may find voice AI socially attractive and satisfying.

Taken together, this study investigates the relationships between social loneliness and individuals’ intentions to continue using voice AI by proposing two mediation pairs: (1) privacy concerns and satisfaction and (2) social attraction and satisfaction. Users’ perceptions are specifically expected to mediate the relationship between loneliness and usage intention. Lonely people will presumably (a) express fewer privacy concerns (mediator 1 in pair 1) when using voice AI, and (b) see their device as a more socially attractive actor (mediator 1 in pair 2), leading to greater satisfaction (mediator 2 in both pairs). These associations should then enhance one’s likelihood of continuing to use voice AI. Prior research has explored influential variables from the angle of AI and user motives [15,16,17]. In the AI context, little research has looked into the role of user perceptions as mediators. To fill this gap, the present study takes a psychological approach to examine how users’ loneliness are associated with their perceptions (privacy and social attractiveness of AI) and these associations affect satisfaction and behavioral intention. Hypotheses are therefore put forth:

**Hypothesis** **1 (H1).** *Social loneliness is positively related to one’s intentions to continue using voice AI.*

**Hypothesis** **2 (H2).** *Social loneliness is positively related to (a) privacy concerns, (b) voice AI’s social attractiveness, and (c) satisfaction.*

**Hypothesis** **3 (H3).** *(a) Privacy concerns, (b) voice AI’s social attractiveness, and (c) satisfaction are positively related to one’s intentions to continue using voice AI.*

**Hypothesis** **4 (H4).** *(a) Privacy concerns and (b) voice AI’s social attractiveness are positively related to satisfaction.*

**Hypothesis** **5 (H5).** *Privacy concerns and satisfaction serially mediate the relationship between social loneliness and one’s intentions to continue using voice AI.*

**Hypothesis** **6 (H6).** *Voice AI’s social attractiveness and satisfaction serially mediate the relationship between social loneliness and one’s intentions to continue using voice AI.*

**Figure 1:**
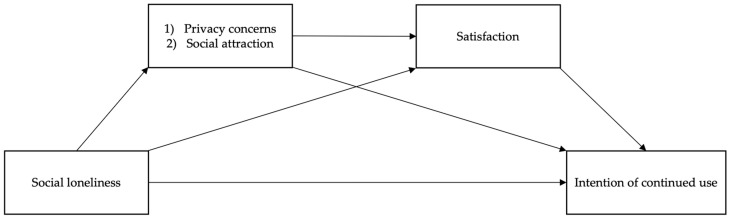
Proposed research model.

Our proposed research model is displayed in Figure 1.

## 2. Materials and Methods

### 2.1. Sample and Procedure

An online survey was conducted in this study. Current voice AI users were recruited through a research firm and offered incentives for survey completion. Additional steps were executed to ensure the eligibility of respondents to be part of this study. Prospective respondents were directed out of the survey if they stated they had used a voice AI device for either less than 1 month (i.e., duration of ownership) or “almost never” (i.e., frequency of device use). Only qualified respondents continued to take part in this survey. Eligible respondents gave consent to participate and were first asked to report which voice AI device they used (e.g., Google Home). They next completed measures regarding social loneliness. Then, they were asked to consider their voice AI device with respect to privacy concerns, social attractiveness, satisfaction, and continued usage intentions. Respondents also answered a series of questions on their device usage behavior and demographics. They were finally debriefed and thanked for their participation.

The initial sample consisted of 307 participants. After eliminating responses that included an inappropriate voice AI, were incomplete, or contained extreme or abnormally consistent response patterns, the final sample included 292 respondents (56.8% female). Respondents were between 18 and 63 years old (*M* = 24.17, *SD* = 8.25). Approximately half were White/Caucasian (53.4%), followed by 16.1% Hispanic, 13% Black/African American, and Asian/Asian American. Roughly 35% reported having owned and used their voice AI for 1 year or longer. Most respondents (63.4%) used voice AI daily or several times a day. Table 1 presents a sample profile.

### 2.2. Measures

All survey items were drawn from established instruments. Items were scored on a 7-point Likert scale (1 = strongly disagree, 7 = strongly agree) unless indicated otherwise. A single index was generated by averaging the item scores for each measure. All measures were reliable (Cronbach’s *α* ≥ 0.80).

Loneliness was measured based on Hughes et al.’s [28] shortened 3-item loneliness scale (*α* = 0.95). Respondents were asked to indicate the extent to which they felt an absence of connectedness with others. This scale has been used elsewhere to assess loneliness in relation to adoption of digital communication technology [29].

Six items (*α* = 0.93) on privacy concerns addressed respondents’ degree of worry about their personal privacy when using voice AI [26]. The scale’s items were slightly revised to fit the context of voice AI devices. Note that a higher mean value on this measure signified lower privacy concerns.

Social attraction of voice AI concerns the extent to which users perceive conversation with voice AI positively, as they do with others. This construct was measured using 4 items (*α* = 0.89) [27]. Previous literature has used this measure in the AIBO context. To suit the context of the present research, AIBO was modified to include voice AI devices.

A 4-item satisfaction measure (*α* = 0.83) [15,30] assessed users’ gratification with voice AI.

An individual’s intention of continued use of voice AI was adopted from Choi and Drumwright’s research [15] and measured with 3 items (*α* = 0.82). The participants were asked their intention to keep using the voice AI assistant in the future.

The details of the measures are in Appendix A.

### 2.3. Data Analysis

Per Hayes’ recommendation, partial correlations were examined between the mediation pairs (i.e., privacy concerns and satisfaction; social attraction and satisfaction) [31]. For hypothesis testing, a serial mediation model was used with PROCESS Macro Model 6 (bootstrapping *m* = 5000; mean-centered for construction of products).

## 3. Results

### Hypotheses Testing

The results showed that the initial correlation between privacy concerns and satisfaction was significant (*r*(290) = 0.475, *p* < 0.001) and remained significant when controlling for loneliness (*r*(290) = 0.456, *p* < 0.001). The same pattern applied for social attraction and satisfaction (initial correlation: *r*(290) = 0.373, *p* < 0.001; when controlling for loneliness: *r*(290) = 0.325, *p* < 0.001).

The association between loneliness and continued usage intention was not significant for privacy concerns (B = 0.06, standard error [SE] = 0.02, *p* = 0.15) or social attraction (B = 0.07, SE = 0.02, *p* = 0.09). H1 was thus not supported. Loneliness was positively related to privacy concerns (B = 0.16, SE = 0.05, *p* < 0.01), social attraction (B = 0.33, SE = 0.05, *p* < 0.001), and satisfaction (privacy concerns: B = 0.16, SE = 0.03, *p* < 0.01; social attraction: B = 0.12, SE = 0.03, *p* < 0.05). Therefore, H2a–c were supported. Neither privacy concerns (B = 0.03, SE = 0.03, *p* = 0.56) nor social attraction (B = −0.04, SE = 0.03, *p* = 0.30) were significantly associated with continued usage intention, failing to support H3a,b. H3c was supported: satisfaction was positively related to this intention (privacy concerns: B = 0.74, SE = 0.05, *p* < 0.001; social attraction: B = 0.76, SE = 0.04, *p* < 0.001). Satisfaction also mediated the association between loneliness and continued usage intention (privacy concerns: 95% CI (Confidence interval): [0.023, 0.105]; social attraction: 95% CI: [0.003, 0.095]). Privacy concerns (B = 0.45, SE = 0.03, *p* < 0.001) and social attraction (B = 0.34, SE = 0.04, *p* < 0.001) each displayed significantly positive relationships with satisfaction, supporting H4a,b.

Lastly, serial mediating effects were confirmed as well. The indirect effect of loneliness on continued usage intention through privacy concerns and satisfaction was significant (95% CI: [0.005, 0.055]). H5 was hence supported. Social attraction and satisfaction jointly mediated the impact of loneliness on intention (95% CI: [0.026, 0.074]), lending support to H6. Indirect effects are summarized in Table 2 and Table 3.

## 4. Discussion

Findings in this study showed that users’ perceptions of voice AI influenced the relationship between respondents’ loneliness and continued usage intentions. Specifically, privacy concerns, social attraction, and satisfaction demonstrated full serial mediations because loneliness had no direct effects on intention when mediators were present. These findings echo earlier studies [26] and reinforce the importance of privacy protection in the voice AI context, especially for user satisfaction and sustainment. Transparency about privacy regarding voice AI use appears paramount since it is a continuously argued topic in both academia and industry. Ensuring alleviated privacy concerns by offering transparent data use may be necessary to provide a better user experience by letting users disclose themselves [32]. Lonely users also seemed to perceive voice AI as an attractive social agent and to be satisfied using it. These findings enrich CASA studies indicating social interaction as a strong motivator for smart speaker adoption with effects on behavioral intention [15,22]. Voice AI’s social presence may give lonely users the sense that they are speaking with a person. This experience can be gratifying and inspire continued device use [2,22].

This study found evidence supporting the sociopsychological influences of loneliness and users’ perceptions of voice AI on behavioral intention. Findings add depth to our understanding of voice AI. Lonely individuals continued using voice AI thanks to its attractiveness as a conversation partner, low privacy concerns, and satisfactory usage experiences. In supplementing research showing that routine conversation eases loneliness [33], this study suggests that voice AI could represent an emerging resource, as a technology-based device, for people in this state. The findings of this research provide additional empirical support for motivational research in AI-related fields by attesting to psychological factors that can explain aligning with users’ motivation in terms of using AI technology. The confirmed serial mediation effects paint a richer picture of how the perceived privacy and social features of voice AI inform users’ satisfaction and behavioral intentions.

Aside from the theoretical values, this study’s results also offer practical implications. Privacy and social attraction were identified as key aspects of users’ satisfaction and device use. Device designers should thus integrate more elaborate privacy options and conversational functions [34]. For instance, voice AI could include a screen and an option to use a human name instead of wake words [35]. Moreover, this research provides useful implications for health professionals. Regarding mental health, technological devices have been recommended for reducing loneliness [9,36]. Clinicians or managers of senior living homes could use voice AI to facilitate patients’ communication [36,37]. In addition to senior living, in an industrial context, hospitals and health apps could leverage AI as a gadget for managing medical records, offering virtual health assistants, and tracking mental and physical health conditions [38]. Understanding what factors make people maintain use of voice AI devices can enable practitioners in industries to enhance patients’ healthier life and the effectiveness of health services through such devices.

Several limitations of this research leave room for future exploration. Age-related effects were not considered. Gen Z is known to be lonely [8]. Subsequent work can replicate the study to examine generational differences. For example, digital natives (e.g., Gen Z) and digital immigrants (e.g., Gen X) could diverge in terms of privacy concerns considering their different attitudes toward technology [39]. Scholars could examine how members of these two generations with feelings of loneliness perceive AI devices and form their usage behaviors. Based on the results of academic research, industrial practitioners may then be encouraged to design voice AI devices providing more details of privacy protection using easy languages for older generations. Another potential avenue for research is investigating the sociopsychological impacts of voice AI. This research’s focus is on device-related consequences (i.e., intention to continue use of voice AI). Researchers should further ponder the potential of voice AI as a substitute for physical social interaction. This role may influence psychological well-being by reducing loneliness and increasing happiness [6]. Further examination using qualitative data, such as from diaries or in-depth interviews, could shed greater light on contextual factors of voice AI use as well.

## Figures and Tables

**Table 1:** Sample profile.

Age, Mean (min.–max.)	24.2 (18–63) Years		
Gender	Male (42.5)	Ethnicity	White/Caucasian (53.4)
	Female (56.8)		Hispanic (16.1)
	Prefer not to say (0.7)		Black/African American (13.0)
			Asian/Asian American (13.0)
			Other (4.5)
Usage frequency	Several times a day (28.1)	Ownership period	1 year or longer (34.9)
	Daily or almost daily (35.3)		6 months to less than 1 year (37.4)
	At least weekly (25.0)		1 to less than 6 months (27.7)
	At least monthly (11.6)		

Note. Values are expressed in percentages, except age.

**Table 2:** Indirect effects of loneliness through mediator pair 1 on intention.

Effects	B	SE	Bootstrapping CI	
			Lower	Upper
Loneliness → Privacy concerns → Intention	0.002	0.004	−0.005	0.012
Loneliness → Satisfaction → Intention	0.064	0.021	0.023	0.105
Loneliness → Privacy concerns → Satisfaction → Intention	0.029	0.013	0.005	0.055

Note. B = standardized beta; SE = standard error; CI = 95% Confidence Intervals.

**Table 3:** Indirect effects of loneliness through mediator pair 2 on intention.

Effects	B	SE	Bootstrapping CI	
			Lower	Upper
Loneliness → Social attraction → Intention	−0.008	0.008	−0.027	0.007
Loneliness → Satisfaction → Intention	0.049	0.023	0.003	0.095
Loneliness → Social attraction → Satisfaction → Intention	0.047	0.013	0.026	0.074

Note. B = standardized beta; SE = standard error; CI = 95% Confidence Intervals.

## Data Availability

The data of this study are available upon reasonable request.

## Appendix A. Items of Measurement

**Measurement Items**
Loneliness (α = 0.95)
In general, I feel like I lack companionship
In general, I feel like I am often left out of social situations
In general, I feel isolated from others
Privacy concerns (α = 0.93)
I feel that the voice AI assistant protects my personal privacy
I feel that my transactions are safe when I am shopping using the voice AI assistant
The voice AI assistant has an adequate number of security features
I feel like my privacy is protected when I am using the voice AI assistant
I trust that the voice AI assistant will not misuse my personal information
I trust that this voice AI assistant will not provide my information to others sites without my permission
Social attraction of voice AI (α = 0.89)
I think I could have a good time with my voice AI device
I think my voice AI device could be a friend of mine
I would enjoy a casual conversation with my voice AI device
I would like to spend more time with my voice AI device
Satisfaction (α = 0.83)
Overall, I am satisfied with my voice AI device
Overall, interacting with a voice AI device is emotionally satisfying and pleasant
Overall, the functions provided by a voice AI device meet my needs
I am satisfied with my decision to purchase a voice AI device
Intention of continued use of voice AI (α = 0.82)
I plan to keep using a voice AI device in the future
I want to continue using a voice AI device
I intend to recommend a voice AI device to my friends
